# Paravertebral retroperitoneal ancient schwannoma mimicking irritable bowel syndrome

**DOI:** 10.1093/jscr/rjae283

**Published:** 2024-05-05

**Authors:** Venkiteswaran Muralidhar, Chandrasekaran Kundhavai, Ramvivek Modiem, Singaram Sowmya

**Affiliations:** Department of Bioinformatics, University of Birmingham, Birmingham B15 2TT, United Kingdom; Chettinad Academy of Research and Education, Kelambakkam, Chennai 600103, India; Chettinad Academy of Research and Education, Kelambakkam, Chennai 600103, India; Chettinad Academy of Research and Education, Kelambakkam, Chennai 600103, India; Chettinad Academy of Research and Education, Kelambakkam, Chennai 600103, India

**Keywords:** retroperitoneal, paravertebral, ancient, schwanomma

## Abstract

We report a case of paravertebral retroperitoneal ancient schwannoma (RPAS) with symptoms suggestive of irritable bowel syndrome that was relieved after resection. Very few cases have been reported of RPAS with gastrointestinal symptoms. Increased bowel activity associated with RPAS has not been reported. Our case report suggests that RPAS may present with increased bowel frequency that could be relieved after surgical resection.

## Introduction

We report a case of retroperitoneal ancient schwannoma (RPAS) arising paravertebrally from the ventral rami of the second lumbar nerve. The symptoms were mimicking that of an irritable bowel syndrome [1], which was relieved following resection of the tumour. There are many case reports and reviews of RPAS [2–6]. However, extremely few cases of RPAS that presented with gastrointestinal symptoms have been reported [2, 7–9]. Around 1% of schwannomas occur in the retroperitoneum and less than 1% of soft tissue tumours are ancient schwannomas [10]. Most RPAS are asymptomatic, have typical histopathology, are treated by resection and rarely become malignant [11, 12]. The differential diagnosis is listed in Table 1 [12].

**Table 1:** Differential diagnosis of paravertebral retroperitoneal schwannoma [12].

Neural Origin	Schwannoma
	Gangioneuroma
	Meningioma
	Neurofibroma
	Ependymoma
Neuroendocrine	Paraganglioma
Vascular	Arteriovenous Malformations
	Aneurysms
Malignancies	Soft tissue sarcomas
	Secondary Metastasis
	Osteosarcoma
Miscellaneous	Tuberculous Cold Abscess
	Prolapsed Intervertebral disc
	Haematoma

## Case report

**Figure 1:**
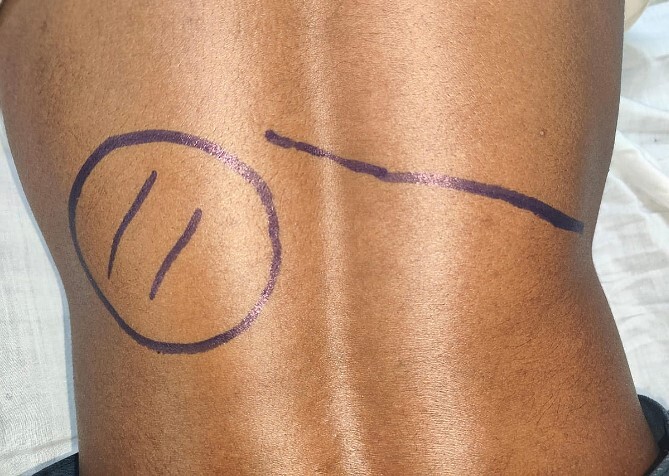
Clinical photograph showing a fullness in the left loin (cross marked within an oval). The line on the right side shows the location of the 12th rib on that side.

**Figure 2:**
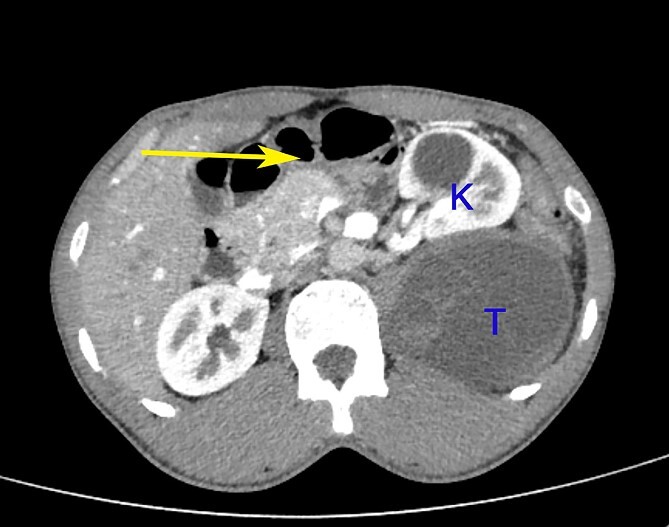
A contrast-enhanced CT scan of the abdomen at the level of the L2 vertebra shows a paravertebral tumour (T) posterior to the kidney (K). The tumour shows peripheral enhancement and inhomogeneous intra-tumoural contrast enhancement. The left colon is pushed to the right and anteriorly (arrow).

**Figure 3:**
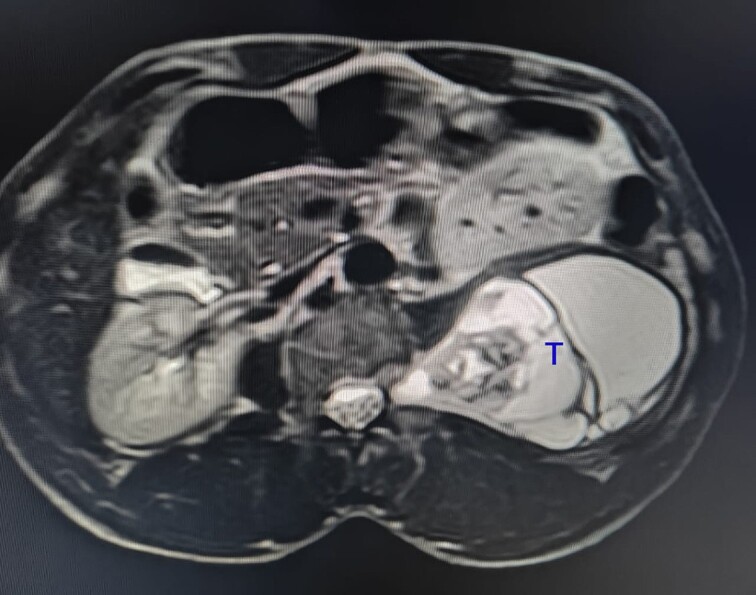
The tumour (T) shows a high-intensity signal on a T2-weighted MRI.

**Figure 4:**
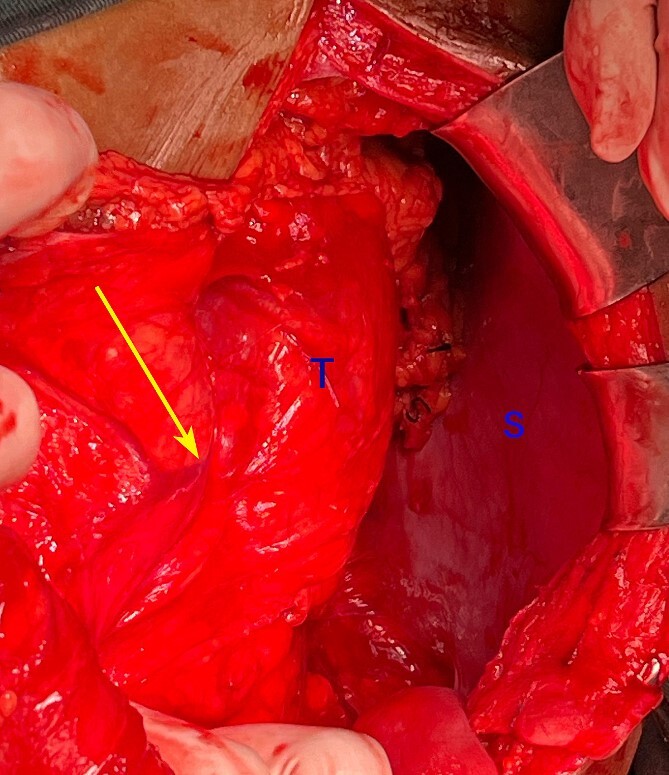
Intraoperative photograph showing the left colic vessels are splayed (arrow) by the tumour (T). S: spleen.

**Figure 5:**
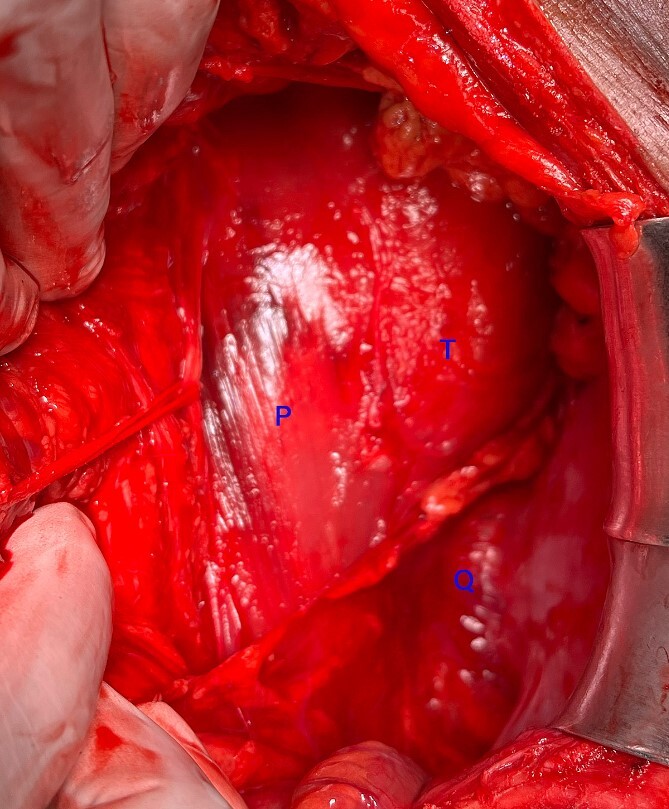
Intraoperative photograph showing the paravertebral location of the tumour posterior to the sympathetic chain (looped with an umbilical tape). T: tumour, P: Psoas, Q: quadratus lumborum.

**Figure 6:**
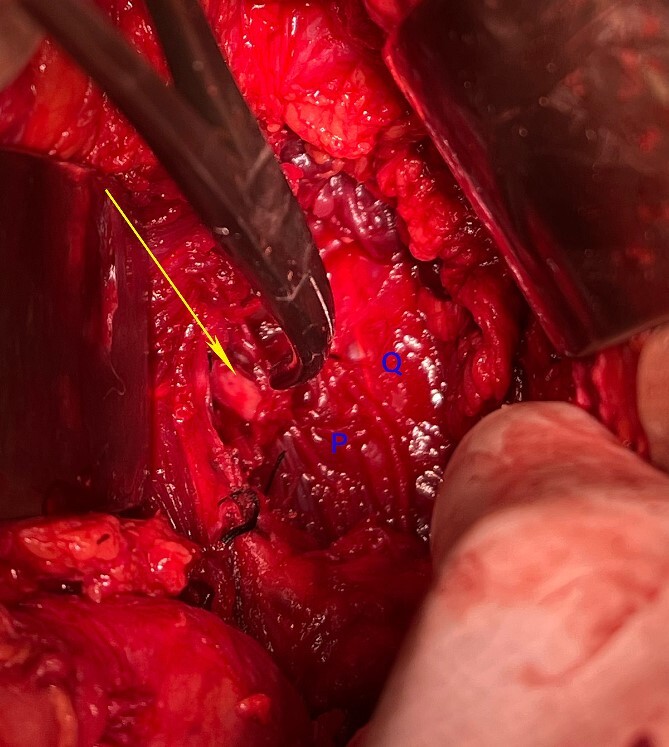
Intraoperative photograph after the tumour resection showing the cut end of the second lumbar nerve (arrow). P: Psoas Major, Q: quadratus lumborum.

**Figure 7:**
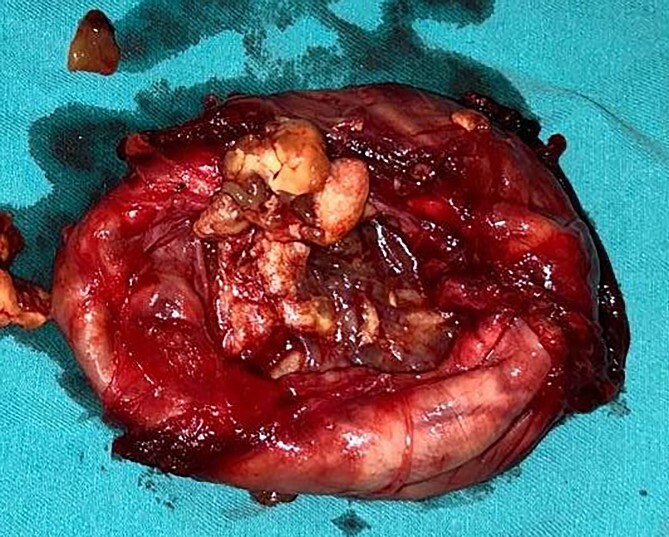
Photograph of the excised tumour showing cystic areas and areas of haemorrhages.

**Figure 8:**
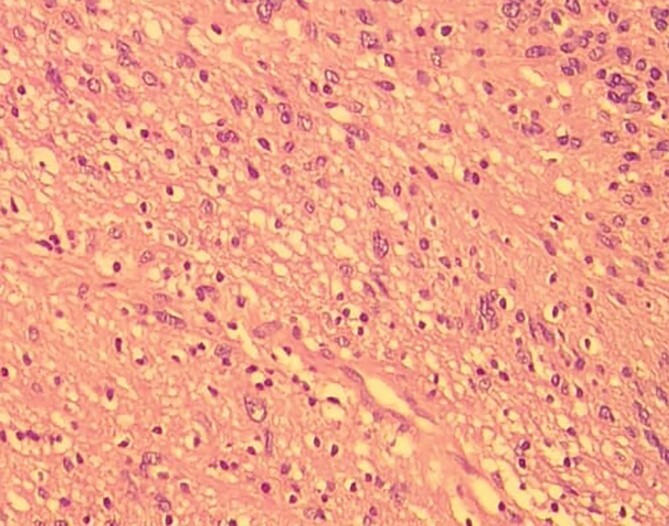
Low-resolution 200x H&E histopathology image of the tumour showing hypercellular (Antony A) areas.

**Figure 9:**
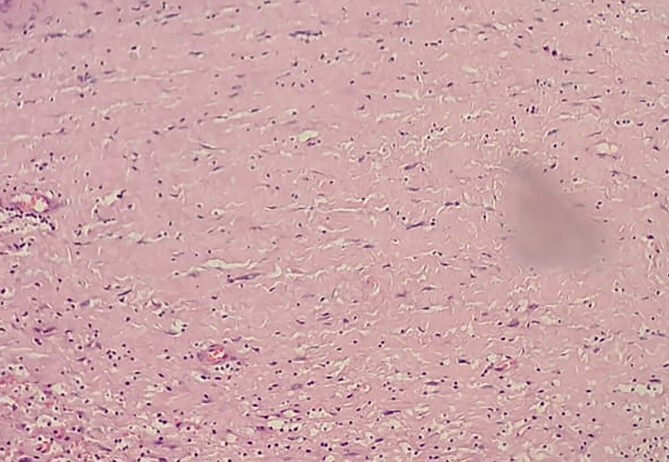
Low-resolution 200x H&E histopathology image of the tumour showing hypocellular (Antony B) areas.

**Figure 10:**
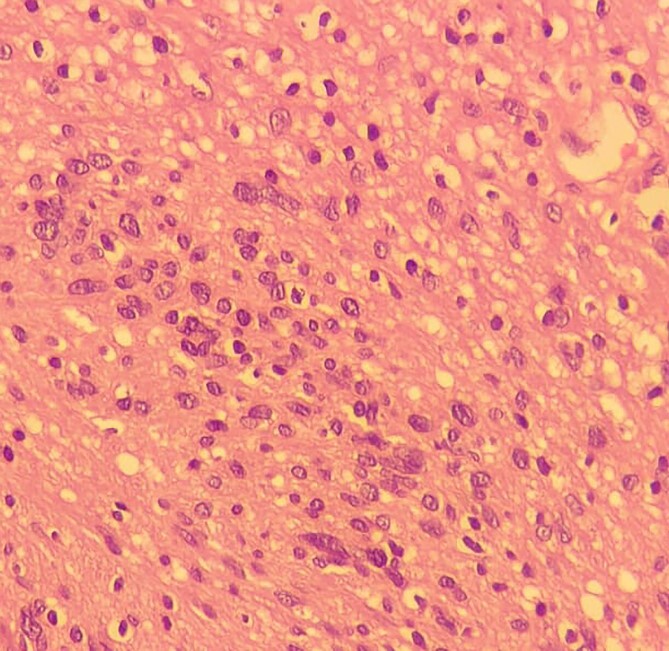
High-resolution 400x H&E histopathology image of the tumour showing nuclear atypia suggestive of ancient schwannoma.

**Figure 11:**
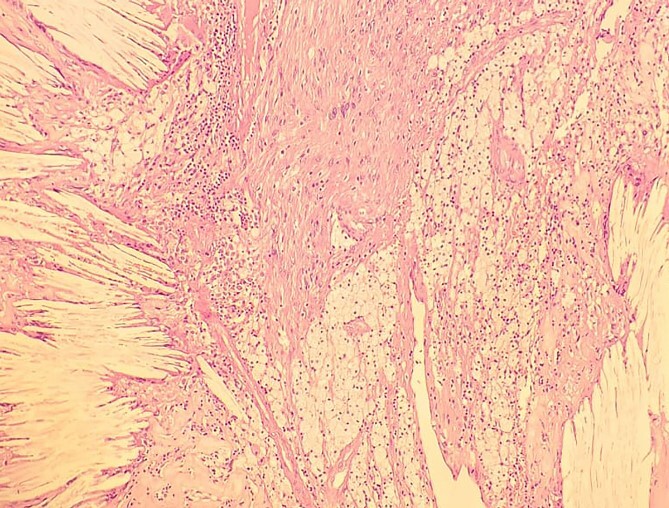
Low-resolution 200x H&E histopathology of the tumour showing foamy macrophages and cholesterol clefts suggestive of degenerative changes, suggestive of ancient schwannoma.

**Figure 12:**
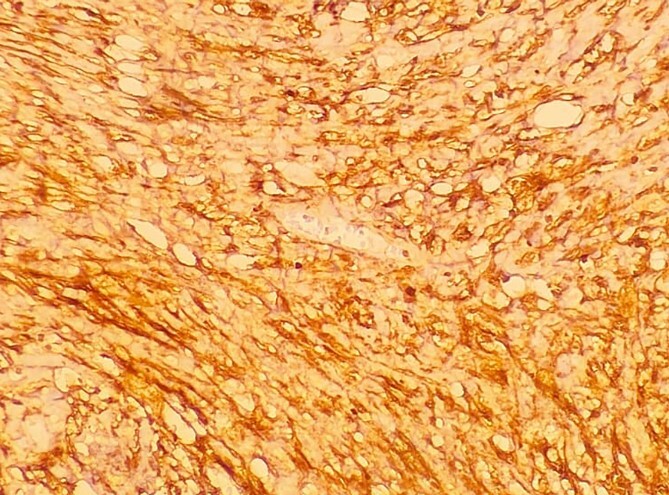
A low-resolution 200x S-100 immunostained histopathology image of the tumour showed strongly positive staining.

A man in his 30s presented with symptoms suggestive of irritable bowel symptoms with increased bowel movements coinciding with the onset of colicky abdominal pain for the past 8 months. The bowel movements relived his pain and had no remission from his symptoms. He had no relevant medical comorbidities in the past. The patient had normal vitals. There were no physical exam abnormalities except for a slight fullness in the left renal angle (Fig. 1). There was nothing significant on a rectal examination. An X-ray of the chest showed normal findings. Blood investigations showed: haemoglobin 130 g/l, total leukocyte: 7.4 × 10^9^/l, serum creatinine 0.52 mg/dl, fasting blood sugar 90 g/dl, alanine transaminase 26 U/L, aspartate transaminase 42 U/L, alkaline phosphatase 32 U/L and total bilirubin 0.8 mg/dL. Ultrasonography showed a 10-cm mass, with mixed echogenicity. Posterior to the left kidney. Colonoscopy was normal. An abdominal CT scan showed a paravertebral tumour with peripheral enhancement and heterogenous contrast within the tumour (Fig. 2). The tumour showed high intensity on a T2-weighted MRI (Fig. 3). The left kidney and colon were displaced anteriorly. Fine-needle aspiration biopsy was inconclusive. The clinical diagnosis was a retroperitoneal schwannoma. On laparotomy, the left colic vessels appeared to be splayed by the tumour (Fig. 4). The tumour 11 × 6 × 3 cm^3^ (Fig. 5), which seems to be arising from the L2 nerve, was resected completely (Fig. 6). The patient had no sensory or motor loss postoperatively. He was discharged without complications on the 10th postoperative day. His colonic symptoms had disappeared after surgery. The gross appearance of the resected tumour showed cysts and haemorrhage patches (Fig. 7). Histopathology showed areas of hypercellularity (Antony A) and hypocellularity (Antony B) with degenerative changes leading to nuclear atypia and cystic spaces, typical of an ancient schwannoma (Figs 8–11). Immunohistochemistry with S-100 was positive (Fig. 12). He was devoid of digestive symptoms or radiological evidence of recurrence at 12 months.

## Discussion

There are three reports of RPAS who presented with constipation that were relieved after resection [2, 7, 9]. In another case, a patient presenting with endoscopically proven colitis had RPAS that was relieved after resection [8]. The authors’ hypothesized that vascular congestion of the colon could have been a reason for the association of colitis with RPAS [8]. Indeed, in our case, the colic vessels were splayed (Fig. 4). We report for the first time a case of increased bowel movements mimicking irritable bowel syndrome that was relieved after resection of the RPAS. The lack of remission of the patient’s lower abdominal symptoms precludes a definitive diagnosis of IBS.
